# Exercising in the Fasted State Reduced 24-Hour Energy Intake in Active Male Adults

**DOI:** 10.1155/2016/1984198

**Published:** 2016-09-21

**Authors:** Jessica L. Bachman, Ronald W. Deitrick, Angela R. Hillman

**Affiliations:** ^1^University of Scranton, 237 Jefferson Ave., Scranton, PA 18510, USA; ^2^Marywood University, 2300 Adams Ave., Scranton, PA 18509, USA

## Abstract

The effect of fasting prior to morning exercise on 24-hour energy intake was examined using a randomized, counterbalanced design. Participants (12 active, white males, 20.8 ± 3.0 years old, VO_2max_: 59.1 ± 5.7 mL/kg/min) fasted (NoBK) or received breakfast (BK) and then ran for 60 minutes at 60%  VO_2max_. All food was weighed and measured for 24 hours. Measures of blood glucose and hunger were collected at 5 time points. Respiratory quotient (RQ) was measured during exercise. Generalized linear mixed models and paired sample *t*-tests examined differences between the conditions. Total 24-hour (BK: 19172 ± 4542 kJ versus NoBK: 15312 ± 4513 kJ; *p* < 0.001) and evening (BK: 12265 ± 4278 kJ versus NoBK: 10833 ± 4065; *p* = 0.039) energy intake and RQ (BK: 0.90 ± 0.03 versus NoBK: 0.86 ± 0.03; *p* < 0.001) were significantly higher in BK than NoBK. Blood glucose was significantly higher in BK than NoBK before exercise (5.2 ± 0.7 versus 4.5 ± 0.6 mmol/L; *p* = 0.025). Hunger was significantly lower for BK than NoBK before exercise, after exercise, and before lunch. Blood glucose and hunger were not associated with energy intake. Fasting before morning exercise decreased 24-hour energy intake and increased fat oxidation during exercise. Completing exercise in the morning in the fasted state may have implications for weight management.

## 1. Introduction

Manipulating dietary intake can be beneficial for athletes and active individuals striving towards improvements in fitness and body composition [1]. Improvements in weight management are a common motivator for individuals to begin and adhere to regular exercise [2]. Competitive athletes also focus on weight as body composition may have an impact on an athlete's cardiorespiratory fitness, strength, agility, overall performance, and appearance [1]. Energy balance, the relationship between energy consumed and energy expended, is a simple equation on paper but is a complicated concept in practice that ultimately determines whether an individual's weight increases, decreases, or stays the same [3]. Exercise has been found to alter energy balance by increasing energy expended as well as by modifying energy intake [4, 5]. Research has shown that, following a single bout of exercise, individuals will typically either reduce energy intake acutely or wait an extended time before initiating food consumption, leading to a short-term energy deficit [5–7]. While some researches suggest that this acute energy shortage may help produce a negative energy balance conducive for weight loss [8], others suggest that individuals compensate for energy expended by eating more later in the day, thus negating any potential benefits for weight loss [9].

A variable that may modify the effects exercise has on energy intake is whether the exercise is completed in a fed or fasted state. Within the past decade training in a state of low carbohydrate availability, often referred to as “training low,” has become a popular technique for athletes and in sports nutrition research [10]. There are a variety of ways by which reducing carbohydrate availability can be achieved. One common protocol is exercising after an overnight fast [11]. While training low has shown some potential physiological benefits in exercise performance [12], the effect of exercise in a fasted state on energy balance is not well understood. Only one study has examined the effect of fasting prior to morning exercise on energy intake[13]. The authors found that there was no difference in energy intake at the meal immediately after exercise but a higher overall energy intake on the days when breakfast was consumed [13]. These results suggest that skipping breakfast prior to morning exercise may aid with reducing overall energy intake. However, this effect has only been examined in one study and the results are limited to only one meal consumed in the laboratory setting immediately after exercise.

Another potential role for fasting in weight and body composition management is that exercising in the fasted state has been shown to alter macronutrient catabolism during exercise. The body relies more on fat as a substrate when exercising in the fasted state [14, 15]. An increase in fat oxidation during exercise could encourage body fat reductions and athletes have reported attempting to reduce body fat by exercising after fasting [16, 17]. Thus, skipping breakfast prior to exercise may potentially help decrease weight and body fat by reducing energy intake and increasing fat oxidation. The purpose of this study was to examine the effect of fasting prior to exercise on the 24-hour energy intake and substrate oxidation during exercise in active males. It was hypothesized that participants would consume less energy and oxidize more fat during exercise on the days they fasted.

## 2. Materials and Methods

### 2.1. Participants

A convenience sample of active men was recruited for this randomized, counterbalanced experimental study through flyers on a university campus in Pennsylvania, United States of America. Inclusion criteria included being male, active [as measured by a minimum VO_2max_ of 43.9 mg/kg/min (50th percentile, American College of Sports Medicine Guidelines, 9th Ed.)] [18], recreational runners (self-reported running at least 30 minutes, 3 times per week for the past month), between 18 and 30 years of age, and usual breakfast consumers (defined as regularly eating breakfast at least 5 times per week). Participants were excluded if they reported a known or suspected wheat or gluten allergy, if they reported having any blood-transmittable diseases, or if they had diabetes mellitus. Twelve participants were recruited for the study to be adequately powered (0.8) to detect an effect size of 0.8 (for differences in total 24-hour energy intake) with the significance level set at 0.05 [19]. Institutional review board approval was obtained prior to initiating any study procedures.

### 2.2. Baseline Measurements

After completing a phone screen, participants completed a demographics questionnaire, written informed consent, and Physical Activity Readiness Questionnaire (PAR-Q) [20]. Baseline measurements included height and weight without shoes and outer layers of clothing. Body density was measured using air displacement plethysmography (BodPod, Life Measurement, Inc., Concord, California, USA) with relative body fat determined using the Siri equation [21]. A treadmill VO_2max_ test was used to confirm aerobic fitness criteria and to determine running speed during the trials.

The VO_2max_ test consisted of a graded treadmill exercise test beginning at 6 miles per hour (mph) and 0% grade, with speed increasing by 1 mph per minute until 9 mph was reached; then grade increased by 1% per min, continuing until the participant reached volitional fatigue. VO_2max_ was confirmed with a plateau in oxygen consumption (not greater than 2 mL/kg/min after an increase in workload), a Rating of Perceived Exertion (RPE) ≥ 18, a Respiratory Exchange Ratio greater than 1.10, or a heart rate at 90% or greater of estimated maximal heart rate (calculated as 220−age) [22]. If a plateau in VO_2_ was not elicited, a majority of the criteria for determining VO_2max_ must have been met. Participants were randomized to trials (Order 1: breakfast trial followed by fasting trial or Order 2: fasting trial followed by breakfast trial) by the researchers picking a number 1 or 2 out of an envelope.

### 2.3. Experimental Design

Each participant completed two trials, one week apart, in this crossover study design. In this study, participants were asked to consume the same foods (using their 24-hour food diary from the day prior to both trials) and avoid strenuous exercise during the 24 hours prior to each trial. Participants were also asked to consume at least 500 mL of water at 8 pm and fast and avoid alcohol and caffeine [23] starting at 10 pm in the evening prior to the trials. When asked as to their compliance with these directives, all participants answered in the affirmative. Participants were blinded to the purpose of the study and did not know which condition they would receive until they arrived at the laboratory.

**Figure 1:**
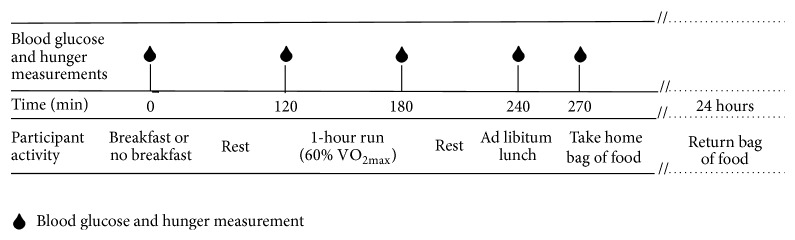
Diagram of participant trials.

Participants arrived at the laboratory and received either breakfast (BK) or no breakfast (NoBK) at 8 am (see Figure 1 for a diagram of the study protocol). Participants in the breakfast trial received a combination of oatmeal and orange juice for breakfast that provided 2 grams of carbohydrate per kilogram of body weight and water ad libitum. The breakfast was high in carbohydrates and low in protein and fat (2298 ± 371 kJ, 82.8 ± 1.5% carbohydrates, 10.2 ± 0.4% protein, and 10.6 ± 1.2% fat) as recommended for a meal 2 hours prior to exercise [1]. Participants were required to consume all of the provided food and at least 500 mL of water. Participants in the fasting trial received water ad libitum, being required to consume at least 500 mL.

At 10 am, participants completed a 1-hour treadmill run at a speed that approximated 60% of their VO_2max_ using the ACSM running equation [18]. After the run, participants received water ad libitum and rested until lunch.

One hour after the completion of the treadmill run, participants were offered lunch at which they were told to eat until they were comfortably full. Lunch consisted of 3 turkey sandwiches (each sandwich was 2 slices of whole wheat bread (86 grams (g)) and turkey (90 g) for all, and participants could choose iceberg lettuce (15 g), tomato (21 g), mustard (2 teaspoons) or mayonnaise (1 tablespoon) which was held consistent between trials), 0.5 bag of Lay's potato chips (170 g), 10 Chips Ahoy chocolate chip cookies (110 g), and apples (450 g) cut into slices with the core removed. The lunch each participant was offered provided 9665 kJ and was 53% carbohydrate, 14% protein, and 33% fat.

After lunch, participants were provided with a bag of food to take home. Participants were asked to eat only foods that were contained in the bag for the remainder of the day and to consume as much or as little of the food as they would like. In the take home bag, participants received 1 box of cooked pasta (454 g) mixed with 1 jar of tomato sauce (680 g), 4 slices of whole wheat bread (172 g), grapes (400 g), 10 Oreo cookies (120 g), and a half gallon of 1% milk (1920 g). The foods in the take home bag provided 19800 kJ and were 65% carbohydrate, 15% protein, and 20% fat. Participants were asked to record their food and beverage intake. All food that was not consumed by the participant was returned to the laboratory on the following morning.

### 2.4. Energy and Macronutrient Intake Assessment

All food provided to participants for 24 hours was weighed (Edlund, E-Series E-160, Burlington, VT, USA) by research staff. The amount consumed was calculated by subtracting the amount remaining from the amount provided and then entered into Nutrition Data Systems for Research version 2014 (University of Minnesota Nutrition Coordinating Center, Minneapolis, MN, USA). Total energy intake was calculated by summing the energy consumed at breakfast (if consumed), buffet lunch, and all food eaten from the take home bag in the evening. “After lunch” energy intake was calculated as the total energy consumed from the foods in the take home bag.

### 2.5. Respiratory Quotient (RQ) and Energy Expenditure Assessment

During the run, expired air was collected (CareFusion, Vmax Encore, Yorba Linda, CA, USA) and the volume of oxygen consumed (VO_2_) and carbon dioxide produced (VCO_2_) were recorded for 5-minute periods from 0–5, 15–20, 35–40, and 55–60 minutes. Nonprotein RQ was calculated as the ratio of carbon dioxide produced to oxygen consumed. Total energy expended during the exercise was calculated from VO_2_ using the Weir equation (1949):

(1) $$\begin{array}{c} \text{kcal}=\left(\;\left(\;1.1\times RQ\;\right)+3.9\;\right)\times {VO}_{2}\;\left(\;L/min\;\right), \end{array}$$

where VO_2_ (L/min) is the absolute O_2_ consumption and protein metabolism was considered to be negligible. This was multiplied by 60 minutes and converted into kJ by multiplying kcal by 4.186.

### 2.6. Blood Glucose and Hunger Assessment

Blood glucose and hunger measurements were taken at five time points: immediately on arrival to the laboratory (arrival), right before exercise (preexercise), within five minutes after exercise (postexercise), right before lunch (prelunch), and right after lunch (postlunch). Glucometers (Bayer Contour 9545C, Bayer HealthCare LLC, Mishawaka, IN, USA) and test strips (Bayer Healthcare LLC, Mishawaka, IN, USA) were used to determine blood glucose. Hunger was measured on a 100 mm Visual Analog Scale anchored with “0, Not hungry at all” and “100, Extremely hungry.” Blood glucose and hunger were measured throughout the trial to potentially help explain any differences in energy intake between trials. Participants were also asked to complete a hunger scale prior to eating each time they ate a meal or snack after they left the laboratory. An average hunger score was calculated for the postlunch eating occasions.

### 2.7. Statistical Analysis

Participant characteristics were examined using means and standard deviations for continuous variables and frequency distributions for categorical variables. The normality of the distribution of data from each variable was assessed using a Shapiro-Wilk test. Data were found to be normally distributed with the exception of the subjective ratings of hunger. Paired sample *t*-tests were used to examine differences in 24-hour, breakfast, lunch, and postlunch energy intake, RQ, energy expenditure, and average postlunch hunger rating between the conditions. Results were considered significant at a minimum of *p* < 0.05. Statistical analyses of blood glucose and hunger were completed using a generalized linear mixed model. Different covariance structures were assumed and the one that minimized the Hurvich and Tsai's criterion was chosen for the final model. Where a significant *F* ratio was observed, post hoc comparisons with Sidak-adjusted *p* values were used to identify which pairs of means were significantly different. Two-tailed statistical significance was accepted at a minimum of *p* < 0.05. Data were analyzed using IBM-SPSS, version 23.

## 3. Results

There were no significant differences in participant baseline demographic characteristics, body composition, or fitness levels between orders (Table 1). There were no significant differences between dietary intake (energy or percentage of energy from macronutrients) recorded on the food records the day prior to each trial (average intake: 10013 ± 3056 kJ, 46.3 ± 12.1% carbohydrate, 18.3 ± 6.1% protein, 33.4 ± 9.5% fat, and 2.0 ± 5.1% alcohol). Metabolic cart data from one trial for one participant was incomplete and thus RQ and energy expenditure analyses include eleven participants rather than twelve.

### 3.1. Energy and Macronutrient Intake

**Figure 2:**
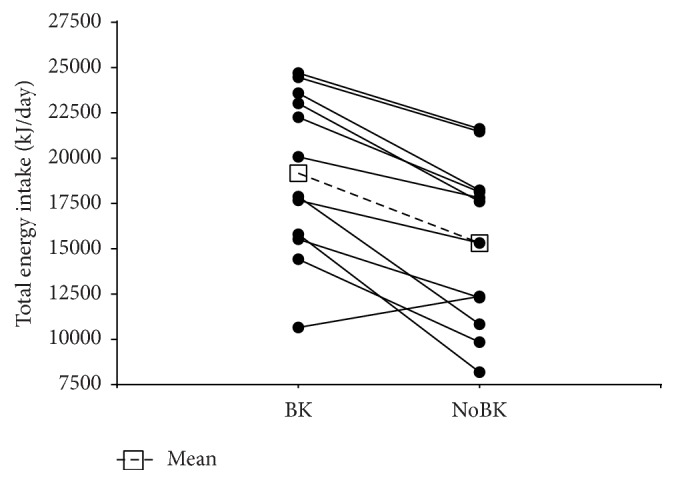
Energy intake for each trial for breakfast (BK) and fasting (NoBK) for each participant.

Total 24-hour energy intake was significantly (*p* < 0.001) higher during the BK trial than the NoBK trial (Table 2). During the BK trial, eleven of the twelve participants consumed more energy over 24 hours (Figure 2). As planned, participants in the BK trial consumed significantly more for breakfast than the NoBK trial. There were no significant differences in energy intake at lunch. After lunch energy intake was significantly (*p* = 0.039) higher during BK than NoBK. There were no significant differences in macronutrient distribution of the foods consumed for the total 24 hours (BK: 67.4 ± 3.5% carbohydrate, 12.9 ± 1.8% protein, and 22.1 ± 4.0% fat; NoBK: 64.8 ± 5.8% carbohydrate, 13.3 ± 2.3% protein, and 24.2 ± 5.6% fat), at lunch (BK: 47.0 ± 5.0% carbohydrate, 15.9 ± 3.4% protein, and 38.9 ± 6.1% fat; NoBK: 45.8 ± 4.3% carbohydrate, 16.7 ± 4.2% protein, and 39.1 ± 6.7% fat), or after lunch (BK: 72.4 ± 3.0% carbohydrate, 12.3 ± 2.3% protein, and 17.8 ± 4.0% fat; NoBK: 73.3 ± 4.7% carbohydrate, 12.2 ± 2.2% protein, and 17.2 ± 4.3% fat) between the conditions.

### 3.2. RQ and Energy Expenditure

During the BK trial, RQ was significantly (*p* < 0.001) higher than that of NoBK. There were no differences between the trials in energy expenditure during the run (Table 2).

### 3.3. Blood Glucose and Hunger

**Figure 3:**
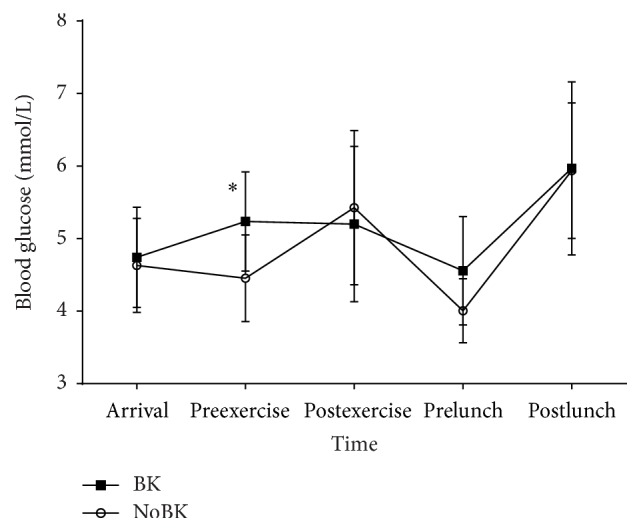
Blood glucose measurements by trial at each time point for breakfast (BK) and fasting (NoBK). ^*∗*^Designating a statistically significant difference between conditions. Preexercise BK had a significantly higher blood glucose than NoBK (*p* = 0.025).

There was a significant (*p* = 0.047) condition by time interaction for blood glucose. The only significant difference between the groups was that prior to exercise BK had a significantly higher blood glucose than NoBK (*p* = 0.025, Figure 3). There was also a significant (*p* < 0.001) main effect for time. In the BK trial, blood glucose was significantly higher after lunch than before lunch (*p* < 0.001). In the NoBK trial blood glucose was significantly higher after lunch than on arrival (*p* = 0.002), before exercise (*p* < 0.001), and before lunch (*p* < 0.001).

**Figure 4:**
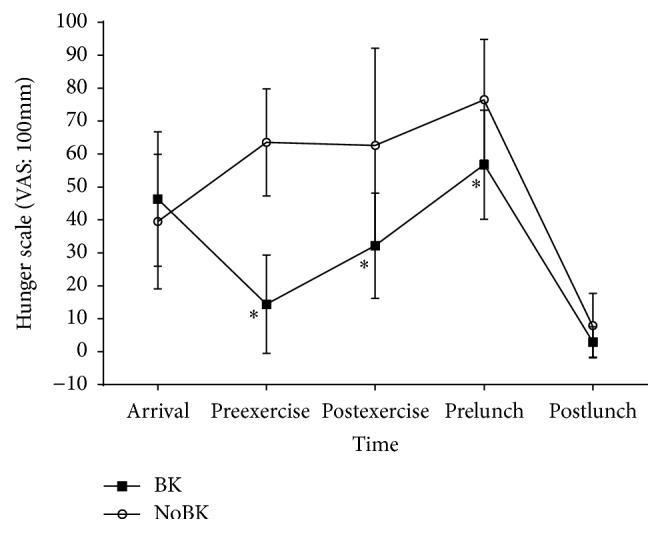
Hunger ratings by trial at each time point for breakfast (BK) and fasting (NoBK). ^*∗*^Designating a statistically significant difference between conditions. Hunger was significantly lower for BK than NoBK before exercise (*p* < 0.001), after exercise (*p* = 0.005), and before lunch (*p* = 0.008).

There was a significant (*p* < 0.001) condition by time interaction for hunger (Figure 4). Hunger was significantly lower for BK than for NoBK before exercise (*p* < 0.001), after exercise (*p* = 0.005), and before lunch (*p* = 0.008). There was also a significant (*p* < 0.001) main effect of time. Hunger was higher on arrival than before exercise (*p* = 0.002) and after lunch (*p* < 0.001), lower before exercise than after exercise (*p* = 0.034) and before lunch (*p* < 0.001), lower after exercise than before lunch (*p* = 0.005), and lower after lunch than after exercise (*p* < 0.001) and before lunch (*p* < 0.001) during the BK trial. During the NoBK trial, hunger was lower after lunch than all other time points (arrival: *p* = 0.002, preexercise: *p* < 0.001, postexercise: *p* < 0.001, and prelunch: *p* < 0.001) and lower on arrival than before lunch (*p* = 0.001). There was also a significant effect of hunger by condition (*p* < 0.001). There were no significant differences in the average hunger for the postlunch eating occasions.

## 4. Discussion

This study examined the effect of fasting prior to exercise on 24-hour energy intake and substrate oxidation in active males. We confirmed our hypothesis that when participants fasted, they consumed less energy over a 24-hour period and relied more on fat as an energy source during exercise. In fact, 11 of the 12 participants consumed less energy on the days they fasted and oxidized more fat during the run. What we did not expect to find was that when participants fasted they also consumed less energy during their evening meals and snacks compared to the days when they ate breakfast. The reduced 24-hour energy intake on fasting days was not only due to the fact that breakfast was skipped but also due to a decreased energy intake at night. This finding suggests that fasting prior to exercise may suppress energy intake over an extended period of time.

With a growing interest in exercising in a state of reduced carbohydrate availability (“training low”), it is important to discover the effects these protocols may have on a variety of physiologic markers. Previous research on training low has found some beneficial improvements in markers that could potentially lead to improvements in athletic performance [10, 11]. Training low can be achieved through a variety of techniques, one of which is exercising in a fasted state. Only one study has examined the effect of fasting prior to exercise on energy intake [13]. This study found that fasting had no effect on energy intake immediately after exercise. The current study also found no effect of fasting on energy intake immediately after exercise but did find suppression in energy intake at the meals and snacks later on in the day. The combination of skipping breakfast and exercising in the fasted state may have an additive effect on suppressing energy intake hours after exercise is completed. It is currently unknown if following this type of protocol over a prolonged period of time could have implications for weight management.

As a means to explain the mechanism behind why fasting may affect daily energy intake, we collected measurements of blood glucose and hunger while the participants were in the laboratory. The glucostatic theory for energy regulation suggests that declines in blood glucose levels will trigger meal initiation and that a meal will continue to be consumed until blood glucose levels reach a preferred range [24–26]. According to this theory, beginning a meal with a lower blood glucose level would promote consumption of a larger volume of food. Our finding of no differences in blood glucose immediately prior to eating lunch may partially explain why there were no differences in energy intake at lunch between the conditions. Our participants were healthy and active young males and that may explain why even with the breakfast manipulation blood glucose remained within normal levels and did not differ between conditions other than right before exercise. An interesting finding of our study was that participants consumed more energy in the evening on the days when they consumed breakfast. Blood glucose was only collected while participants were in the laboratory and, consequently, we are not able to conclude if blood glucose had an effect on energy intake at night.

Hunger may be another factor that can explain differences in energy intake at a meal. It is commonly assumed that the hungrier an individual feels prior to meal consumption, the more energy they will consume at that meal. While this can sometimes be the case, there are many variables that determine energy consumption behaviors such as availability of palatable food, the macronutrient content of the food, and the social and emotional state of the individual that may limit the role of hunger as a predictor of energy intake [27]. Participants in this study reported higher levels of hunger before lunch during the fasting trial but we found no differences in energy intake at lunch, suggesting that hunger did not regulate participant energy intake after exercise. Hunger and energy intake were also not related to the evening meals and snacks as participants reported no differences in the average level of hunger prior to eating even though they consumed significantly more energy in the evenings on the days when breakfast was consumed. Therefore, hunger was not a reliable predictor of energy intake in our study.

In agreement with previous research, [13–15] we indirectly showed that participants used more fat for fuel during exercise after fasting. While some research suggests that the effect of exercise on fat oxidation beyond fuel utilization during exercise is minimal and may have a negligible effect on fat loss over time [14], a recently published study found that the timing of the exercise and the prandial state of participants (fasted versus fed) modulates the effect of exercise on fat oxidation in the postexercise period [28]. This research found that when 60 minutes of continuous exercise was performed in the morning before eating, there was a greater increase in 24-hour fat oxidation than when participants ate prior to exercise or when they exercised later in the day. The increase in fat oxidation was also associated with a greater negative energy balance, suggesting that exercising in the fasted state in the morning may potentially help decrease fat mass and aid weight loss, although longer term studies are needed prior to coming to this conclusion.

Observational studies suggest an association between breakfast consumption and weight such that habitually skipping breakfast is associated with a higher weight and greater weight gain over time [29–31]. However, as summarized in a review by Brown et al., 2013, these findings have not been confirmed by the few randomized controlled trials that have examined this relationship and articles have often misrepresented the causality or the strength of the association [32]. In fact, our findings add to a growing area of research that suggests skipping breakfast may result in a reduced energy intake.

The limitations of this study include the small sample of active males which reduces the generalizability of our results to other populations as well as the application beyond the effects on energy intake for one day. An additional limitation of our study is that substrate oxidation and fuel utilization were only measured during exercise and blood glucose was only measured while participants were in the laboratory and thus we do not know the role these factors may have had on energy intake outside of these times. A strength of this study includes that dietary intake was weighed and measured and did not rely on participant self-report.

Future research examining the effect of skipping breakfast prior to exercise on energy intake in a variety of populations over a longer period of time is needed. Particularly, the potential role of exercising in the fasted state in weight management would be of interest to a broad population. Investigating the mechanism behind the role of breakfast skipping prior to exercise on appetite and energy intake with a particular focus on appetite regulating hormones such as insulin, leptin, and ghrelin could help to explain our findings. Additionally, directly examining a measure of fat utilization as well as examining fuel utilization over an extended period of time could help explain the role fasting before exercise may have on manipulating body composition.

## 5. Conclusions

With the increasing popularity of “training low” protocols that encourage exercising after an overnight fast, it is important that the effects these protocols have on a variety of physiologic components be examined. Particularly, as many athletes and active individuals are interested in improving both physical fitness and body composition, the effect exercising in the fasted state has on energy balance is of interest. Our results add to a growing area of research that suggests that fasting prior to exercise may be advantageous for reducing energy intake and increasing fat oxidation which have potential implications for weight and body composition management.

## Figures and Tables

**Table 1:** Participant baseline demographic and fitness characteristics by order, mean ± SD (*n* = 12).

Participant characteristic	All participants (*n* = 12)	Breakfast first (*n* = 6)	Fasting first (*n* = 6)	*p* value^*∗*^
Age, y	20.8 ± 3.0	20.6 ± 4.1	20.8 ± 1.6	0.928
Average hours run, per week	4.1 ± 2.0	4.3 ± 1.7	4.1 ± 2.5	0.895
Average miles run, per week	19.8 ± 14.7	25.0 ± 19.0	14.8 ± 7.2	0.264
Height, m	1.80 ± 0.06	1.78 ± 0.05	1.82 ± 0.06	0.176
Weight, kg	73.77 ± 9.91	67.95 ± 2.73	79.59 ± 11.27	0.052
Body mass index (kg/m^2^)	22.7 ± 2.6	21.4 ± 1.9	24.0 ± 2.7	0.085
Body fat, %	14.3 ± 3.6	13.6 ± 3.3	15.0 ± 4.1	0.527
VO_2max_, mL/kg/min	59.1 ± 5.7	61.0 ± 5.6	57.1 ± 5.6	0.260

^*∗*^ *p* value is for the differences between the orders.

**Table 2:** Differences in energy intake, respiratory quotient (RQ), and energy expenditure between trials, mean ± SD (*n* = 12 for energy intake data; *n* = 11 for RQ and energy expenditure).

	Breakfast	Fasting	*p* value^*∗*^
Energy intake, kJ			
Breakfast	2298 ± 371	0.0 ± 0.0	*p* < 0.001
Lunch	4609 ± 925	4479 ± 1712	*p* = 0.810
After lunch	12265 ± 4278	10833 ± 4065	*p* = 0.039
Total	19172 ± 4542	15312 ± 4513	*p* < 0.001
RQ during run	0.90 ± 0.03	0.86 ± 0.03	*p* < 0.001
Energy expenditure during run, kJ	3895 ± 492	3732 ± 585	*p* = 0.158

^*∗*^ *p* value is for the differences between breakfast and fasting trial.
